# Discovery of a novel translation-machinery-associated protein that positively correlates with cellulase production

**DOI:** 10.1186/s13068-025-02624-7

**Published:** 2025-02-22

**Authors:** Kexuan Ma, Panpan Zhang, Jian Zhao, Yuqi Qin

**Affiliations:** 1 State Key Laboratory of Microbial Technology, Shandong University, Qingdao, China; 2 National Glycoengineering Research Center, Shandong University, Qingdao, China

**Keywords:** Cellulases, Cycloheximide, Transcription, Transcription factor, Translation, Translation elongation

## Abstract

**Background:**

The production of cellulases by filamentous fungi is a crucial aspect of sustainable bioproduction from renewable lignocellulosic biomass. Following the transcription of cellulase genes in the nucleus, a complex pathway involving translation, folding, and secretion is required to produce extracellular cellulases. Most studies about cellulase production have focused on examining transcriptional regulatory mechanisms and enhancement of enzyme gene levels; comparatively, little is known about protein translation and secretion for cellulase production.

**Results:**

A translation-machinery-associated (TMA) protein PoTma15 was identified in cellulosic *Penicillium oxalicum*. The PoTma15 is conserved in various filamentous fungi, but not in yeast, plants, or animals. All homologous proteins of PoTma15 have previously been uncharacterized. PoTma15 was initially thought to be one of the putative interactors of transcription factor PoXlnR, as it was preyed by tandem affinity purification (TAP) coupled with the mass spectrometry (TAP–MS) technique using PoXlnR as the bait. Subsequent research revealed that PoTma15 is associated with the translation machinery. The top three proteins associated with PoTma15 are orthologs of *Saccharomyces cerevisiae* translation-machinery-associated protein (Tma19), translation elongation factor eIF5A, and ribosomal protein S28, respectively. PoTma15 is widely distributed in fungal hyphae and positively correlates with the production of cellulases and extracellular proteins. Deleting the Po*tma15* gene (Δ*tma15*) decreased cellulase production, while overexpressing the Po*tma15* gene (OE*tma15*) increased cellulase production. However, the Δ*tma15* mutant was not observed to have downregulated transcript levels of major (hemi)cellulase and amylase genes, compared to the *P*. *oxalicum* wild type (WT). The production of extracellular cellulases and extracellular proteins of the Δ*tma15* mutant was less affected by cycloheximide, an inhibitor of eukaryotic translation elongation, compared to the WT strain and OE*tma15* mutant*,* suggesting a stronger resistance to the translation-inhibiting effects of cycloheximide in the Δ*tma15* mutant. The results demonstrate that PoTma15 is a translation-machinery-associated protein that affects translation elongation and, consequently, the production of enzyme proteins.

**Conclusions:**

PoTma15 is the first TMA protein characterized in cellulosic filamentous fungi and the first TMA protein used in fungi to increase cellulase production. PoTma15’s role in the production of cellulases and total extracellular proteins suggests that not only can it be used to widen the cellulase production pathway, but can even be engineered as a target to improve the production of other heterologous protein or bioproducts using filamentous fungi as cell factories in the future.

**Supplementary Information:**

The online version contains supplementary material available at 10.1186/s13068-025-02624-7.

## Introduction

Lignocellulosic biomass is a valuable carbon source for the sustainable production of biofuels and value-added biochemicals. Filamentous fungi such as *Trichoderma*, *Aspergillus*, and *Penicillium* are exceptionally capable of synthesizing and secreting (hemi)cellulases—the necessary lignocellulosic biomass-degrading enzymes that can be used in food, detergent, supplements for livestock, textile and pulp and paper industries, and are critical to sustainable bioproduction based on renewable lignocellulosic biomass [1, 2]. The protein secretion capacity of the famous industrial *Trichoderma reesei* mutant can reach 100 g/l, with up to 80% of cellulases, under appropriate cultivation conditions [3]. *Aspergillus nige*r, *Penicillium oxalicum*, *Myceliophthora thermophila,* and their modified mutants also possess a high capability to synthesize and secrete extracellular (hemi) cellulases [4–7].

(Hemi)cellulase production is mainly controlled at the transcriptional level of enzyme genes. Many transcription factors have been discovered to affect the production of enzyme proteins by activating or inhibiting cellulase gene transcription [8]. For example, transcription factor CreA (Cre1 in *T. reesei*) is a transcriptional repressor of cellulase gene duo to carbon catabolite repression (CCR). The deletion, disruption, or mutation of the *creA*/*cre1* gene can increase (hemi)cellulase production [6, 9]. The transcription activators XlnR (Xyr1 in *T. reesei* and XLR-1 in *Neurospora crassa*), ClrB (Clr2/CLR-2 in *T. reesei* and *Neurospora crassa*), and AraR activate the transcription of (hemi)cellulase genes. Overexpression of the *xlnR*/*xyr1*, *clrB*/*clr2*, or *araR* gene increased the production of extracellular (hemi)cellulases in *T. reesei*, *Aspergillus nidulans*, *P. oxalicum*, or *Neurospora crassa* [10–16]. Point mutations in specific amino acids of XlnR/Xyr1 protein led to high (hemi)cellulase production even in glucose presence, which is a repressing carbon source [17, 18].

Although transcription level underlies the regulation of a wide range of biological processes, translation control has also been found to play roles in fungal growth, cell morphology, conidiogenesis, virulence, and salt tolerance in various fungi such as *N. crassa*, *Magnaporthe oryzae*, and multiple *Aspergillus* spp. [19–24]. The translation of eukaryotic mRNA into polypeptides consists of initiation, elongation, termination, and ribosome recycling, carried out by the translation machinery, primarily including ribosomes, tRNAs, and eukaryotic translation factors such as initiation factors (eIFs), elongation factors (eEFs), and release factors (eRFs) [25]. In addition to translation machinery, some translation-machinery-associated proteins (TMAs) exist that aid ribosome biogenesis, translation initiation, elongation, and ribosome recycling. Most studies about TMA proteins in fungi have been conducted in the eukaryotic model organism *Saccharomyces cerevisiae*. Different TMA protein plays a biological role in the cell. *S. cerevisiae* Tma23 functions in ribosome biogenesis [26]. Tma20/Tma22 heterodimer is responsible for a majority of 40S ribosome recycling events [27]. Tma108 is a ribosome-associated, nascent chain binding factor that binds the N-terminal region of nascent peptides during translation to improve translation efficiency and nascent chain quality control [28].

Eukaryotic transcription and translation occur in the cellular nucleus and the cytoplasmic ribosome, respectively, and are spatially independent processes. While gene transcriptional regulation remains the main focus of most studies on extracellular (hemi)cellulase production, other biological processes, such as translation, secretion, and turnover of secreted enzymes, rather than transcription, might limit a further increase of cellulase production in hyperproducing strains [29]. Modulation of protein translation, folding, and secretory pathways is an efficient intervention for increasing cellulase production, particularly when the transcription of cellulase genes has reached a high level [30, 31]. For example, the simultaneous deletion of transcriptional regulator CxrC and overexpression of translation elongation factor eEF1A significantly increased cellulase and xylanase yields of *P. oxalicum* [32]. An ER-localized sugar transporter, Lac1, is involved in ribosome biogenesis and cellulase production in *T. reesei* [33]. Overexpression of the critical components (Pdi1, Ero1, and BiP) of protein folding and glycosylation-related elements (Gpt1 and Gls2) increased the cellulase production in the engineered *T. reesei* strains [34].

In this study, we identified a previously uncharacterized protein, PDE_06571 (UniProt Entry S7ZSB7), in *P. oxalicum*. This protein is conserved in various filamentous fungi, but not found in yeast, plants, or animals. PDE_06571 was initially thought to be one of the putative interactors of transcription factor PoXlnR. However, subsequent research revealed that it is associated with translation machinery (thus named PoTma15), specifically with translation elongation. PoTma15 is the first TMA protein characterized in cellulosic filamentous fungi and the first fungal TMA protein to be utilized to increase cellulase production.

## Results

### Translation-machinery-associated protein PoTma15 is found in the putative interacting proteins of transcription factor PoXlnR

Most eukaryotic transcription factors do not directly interact with RNA polymerase II to activate or repress transcription when they bind to target genes. Instead, they recruit cofactors such as histone modifiers and chromatin remodeling complexes to regulate transcription through modifications to chromatin structure [35]. For example, transcription repressor Cre1/CreA recruits co-repressor Tup1–Cyc8 complex as the cofactor to repress cellulase gene transcription [36]. The transcription factor XlnR is a well-known transcriptional activator in cellulase-producing fungi. It has been discovered that *T. reesei* TrXyr1 may recruit the Mediator subunit TrGAL11 or chromatin remodeling complex SWI/SNF as the cofactor to facilitate cellulase gene transcription [37, 38]. However, more research is needed to fully confirm TrXyr1's cofactors in cells, as the above results were primarily based on in vitro studies or only a small portion (the activation domain (aa 767–860)) of TrXyr1 protein was studied.

This work initially aimed to capture and identify the cofactors of PoXlnR using tandem affinity purification (TAP) coupled with the mass spectrometry (TAP–MS) technique. TAP–MS is a highly effective method for identifying partners or cofactors of the protein of interest in the research of yeast, *Aspergillus*, *Trichoderma*, and *Penicillium* in vivo [36, 39, 40]*,* as the two-step tandem purification process can significantly reduce the amount of non-specific binding proteins. The *P. oxalicum* XlnR-TAP strain was generated by fusing the TAP tag (HA-FLAG) at the C-terminus of the PoXlnR (PDE_07674, UniProt Entry S7ZQM9). The WT strain 114–2 containing native PoXlnR was used as a control. The biological triplicates were used for the TAP–MS experiment. After two-step tandem purification, the final eluate was divided into three sections: one for SDS-PAGE, one for Western blot, and one for MS–MS assay to identify the putative interacting proteins of the PoXlnR bait, respectively.

**Fig. 1:**
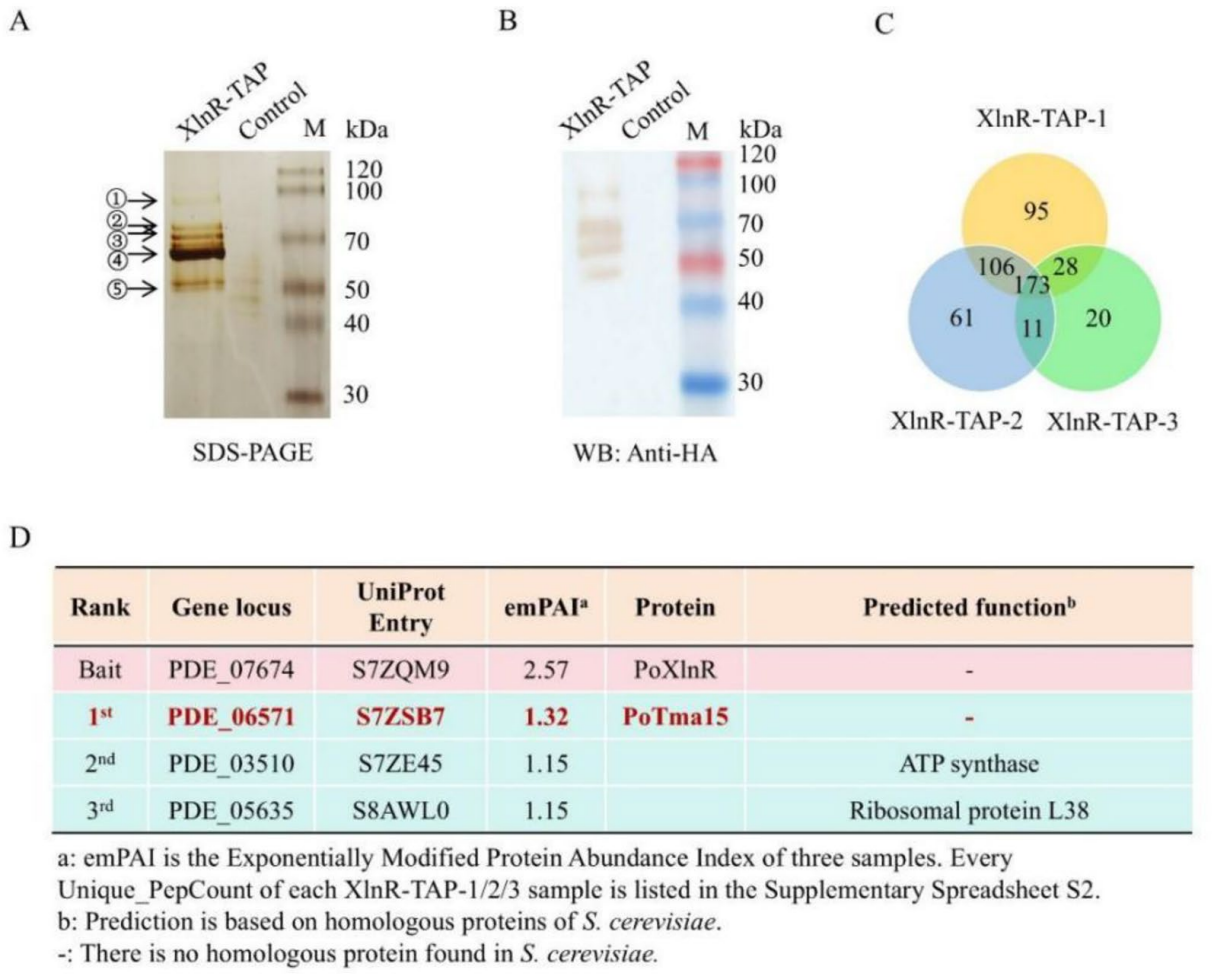
The results of TAP–MS using PoXlnR as bait.** A** Silver staining after SDS-PAGE of the XlnR-TAP eluate. **B** Western blot of XlnR-TAP eluate using anti-HA antibody. **C** Intersection proteins among three XlnR-TAP-1/2/3 biological samples. **D** The top three proteins that have putative interactions with PoXlnR identified by TAP–MS. The detailed information, including Unique_PepCount identified by LC–MS/MS, gene locus, theoretical PepCount, emPAI, and predicted functions of putative interacting proteins, is shown in Supplementary Spreadsheet S3

The theoretical molecular weight of the PoXlnR protein is 107.8 kDa. In addition to a specific band at about 100 kDa (Fig. 1A, black arrow ①,), several other specific bands were observed between the XlnR-TAP and the control from the gel of SDS-PAGE with subsequent silver staining (Fig. 1A). Western blot analysis also indicated the existence of several signals at 50 kDa ~ 100 kDa (Fig. 1B). Five bands (Fig. 1A, black arrows from ① ~ ⑤) were sliced from the gel and identified by LC/MS–MS, respectively. The results of LC/MS–MS are shown in Supplementary Spreadsheet S1. PoXlnR protein was observed in all the results of sliced bands, with the highest score. The results show that the PoXlnR protein has degraded versions.

The third section of the eluent was analyzed by LC–MS/MS to identify the PoXlnR bait and its putative interacting proteins. The PepCount identified in biological triplicates (XlnR-TAP-1, XlnR-TAP-2, and XlnR-TAP-3) through TAP–MS are listed in Supplementary Spreadsheet S2 (Sheets: XlnR-TAP-1/2/3). The proteins were ranked by exponentially modified protein abundance index (emPAI), which was used to estimate protein abundance in the sample [41]. The proteins observed in the controls are listed in Supplementary Spreadsheet S2. The proteins observed in all three XlnR-TAP-1/2/3 samples, but not in the controls are listed in Supplementary Spreadsheet S3 (Sheet: Intersection of three samples). 232, 351, and 402 proteins were identified in the samples XlnR-TAP-1, XlnR-TAP-2, and XlnR-TAP-3, respectively. 173 proteins (including the bait PoXlnR), which were observed in the intersection of three XlnR-TAP-1/2/3 samples, are considered credible proteins that have putative interaction with the PoXlnR (Fig. 1C). The top three proteins are shown in Fig. 1D. As expected, the bait PoXlnR has the highest emPAI among the proteins identified by LC–MS/MS. Among the other proteins, the protein with the highest emPAI is PDE_06571 (Uniprot Entry S7ZSB7).

Therefore, PDE_06571 was initially thought to be one of the putative interactors of transcription factor PoXlnR. However, PDE_06571 was found to be associated with translation machinery in subsequent research. PDE_06571 protein contains 136 amino acids with a molecular mass of 14,666 Da. We name it PoTma15 to facilitate the presentation and understanding. The name is based on the naming scheme used for TMAs according to their molecular mass. For example, the TMA protein in *S. cerevisiae* with a molecular weight of 6,941 Da was named TMA7, and the TMA protein with a molecular weight of 20,278 Da was named TMA20.

### PoTma15 is conserved in filamentous fungi and is widely distributed in fungal hyphae

BLASTP was performed by searching the landmark database (https://blast.ncbi.nlm.nih.gov/smartblast/smartBlast.cgi?), which includes proteomes from 27 genomes of well-studied reference species spanning a broad taxonomic range using the protein sequence of PoTma15 as the query. There is no homologous protein in classic model organisms such as *Escherichia coli*, *Bacillus subtilis*, *S. cerevisiae*, *Schizosaccharomyces pombe*, *Arabidopsis thaliana*, *Caenorhabditis elegans*, *Drosophila melanogaster*, or *Mus musculus*. Although PoTma15 is not found in yeast, plants, or animals, it is phylogenetically conserved and found in various filamentous fungi. The phylogenetic tree indicated the evolution of PoTma15 (Supplementary Fig. S1). PoTma15 exhibits high percent identity of over 80% to homologous proteins of various filamentous fungi such as *Penicillium chrysogenum*, *Aspergillus niger*, *A*. *nidulans*, *N*. *crassa*, *T. reesei*, *Fusarium oxysporum*, *M. oryzae*, and *Metarhizium acridum,* except for its relative low percent identity (32.5%) with homologous protein of *Beauveria bassiana* (Supplementary Spreadsheet S4). All these homologous proteins of PoTma15 have been uncharacterized.

**Fig. 2:**
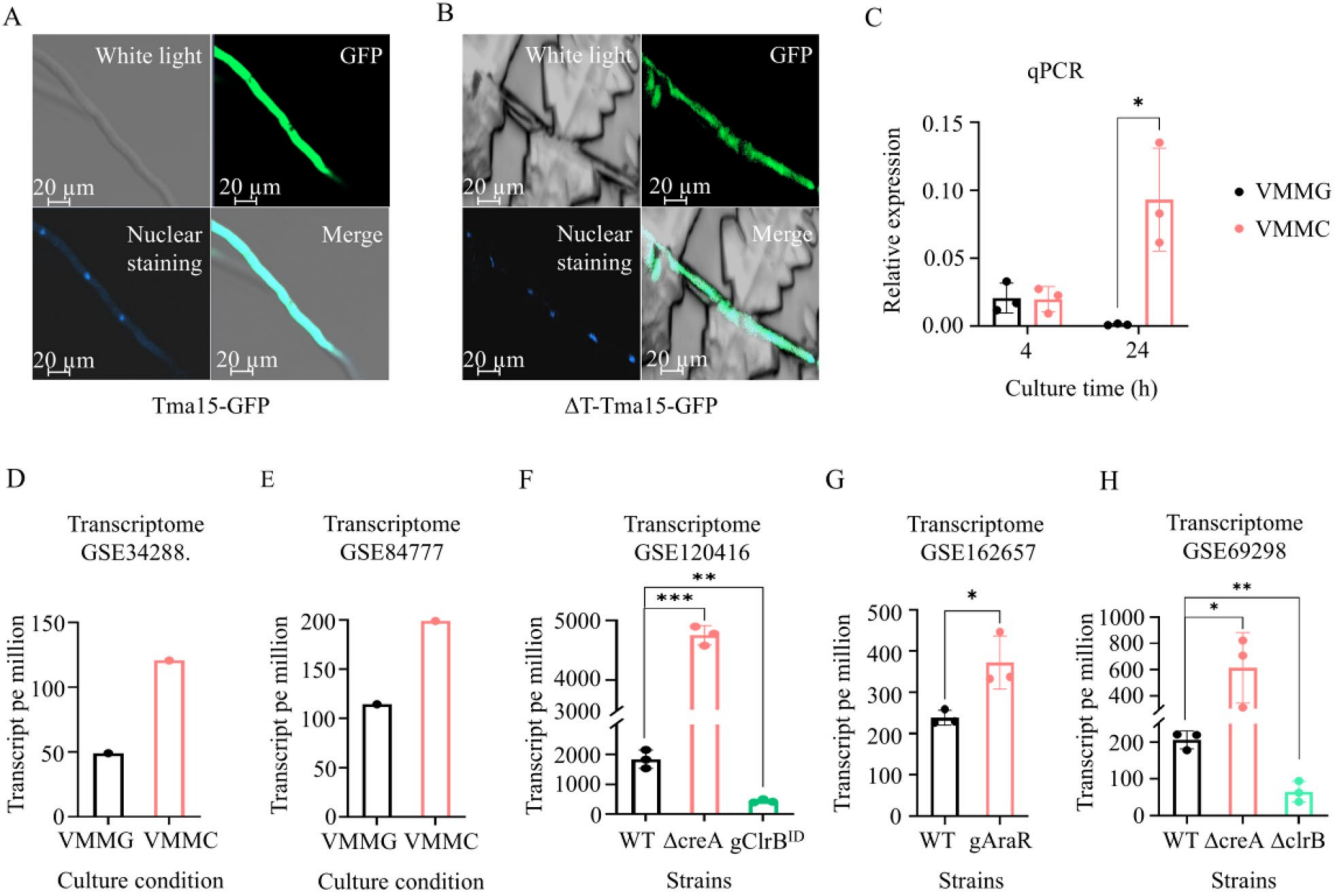
The subcellular localization and transcription levels of PoTma15. The subcellular localization of the PoTma15 protein in mutants Tma15-TAP (**A**) and ΔT-Tma15-GFP (**B**). Upper left, white light; upper right, green fluorescence; bottom left, nuclear staining (blue dots); bottom right, merged. **C** The transcription levels of the Po*tma15* gene analyzed by RT-qPCR. The *P. oxalicum* WT strain was cultivated in VMMG or VMMC medium.** D**–**E** The transcription levels of the Po*tma15* gene analyzed by transcriptome data when the *P. oxalicum* WT strain was cultivated in VMMG and VMMC medium. **F**–**H** The transcription levels of the Po*tma15* gene analyzed by transcriptome data when *P. oxalicum* WT and different mutants were cultivated in the VMMC medium. ΔcreA, the mutant with the *creA* gene deletion. gClrB^ID^, the mutant with the *clrB* gene disruption. gAraR, the mutant with the *araR* gene overexpression. ΔclrB, the mutant with the *clrB* gene deletion. The data of transcriptional profiling were retrieved from the Gene Expression Omnibus (GEO) DataSets of NCBI under the accession number GSE34288 [6], GSE84777 [42], GSE120416 [43], GSE162657 [14], and GSE69298 [12], respectively. *p < 0.05, **p < 0.01, ***p < 0.001

So, what is the biological role of PoTma15 within the cell? Firstly, we studied the subcellular localization of the PoTma15. We fused the PoTma15-coding sequence with the green fluorescent protein (GFP) coding sequence and introduced it to *P. oxalicum* WT to obtain the Tma15-GFP strain. The mutant ΔT-Tma15-GFP (the Tma15 protein fused the GFP in the Δtma15 background) was also constructed. Then, subcellular localization of PoTma15 in strains Tma15-GFP and ΔT-Tma15-GFP was observed (Fig. 2A, B). The results show that PoTma15 forms widespread distribution in the fungal hyphae.

Then, the transcription level of the Po*tma15* gene in the *P. oxalicum* WT was determined using qPCR after the WT strain was cultivated in VMMG and VMMC at 4 h and 24 h. VMMG medium contains glucose, a repressing carbon source inhibiting cellulolytic gene transcription due to CCR, which decreases extracellular cellulases. In contrast, a VMMC medium contains cellulose, an inducing carbon source that activates cellulolytic gene transcription, which increases extracellular cellulases. The qPCR results showed that the transcription of the Po*tma15* gene was remarkably upregulated when VMMC was used as the medium compared to VMMG (Fig. 2C).

In addition to the qPCR results, the transcriptome results also supported that the transcription of the Po*tma15* gene was induced at a low level by cellulose (Fig. 2D, E) [5, 42]. Interestingly, analysis of other transcriptome data of *P. oxalicum* showed that the transcription levels of the Po*tma15* gene were upregulated in mutants with increased cellulase gene expression and cellulase production, such as transcription repressor CreA deletion mutant (ΔcreA) and transcription activator AraR overexpression mutant (gAraR), and downregulated in mutants with decreased cellulase gene expression and cellulase production, such as transcription factor ClrB disruption mutant (clrB^ID^) and ClrB deletion mutant (ΔclrB) (Fig. 2F–H) (12,14,43]. The analysis showed that the transcription of the Po*tma15* gene is positively correlated with the cellulase gene transcription and production, which is consistent with the positive regulation of cellulase gene expression and production by the transcription activator PoXlnR.

### PoTma15 positively correlates with cellulase production

**Fig. 3:**
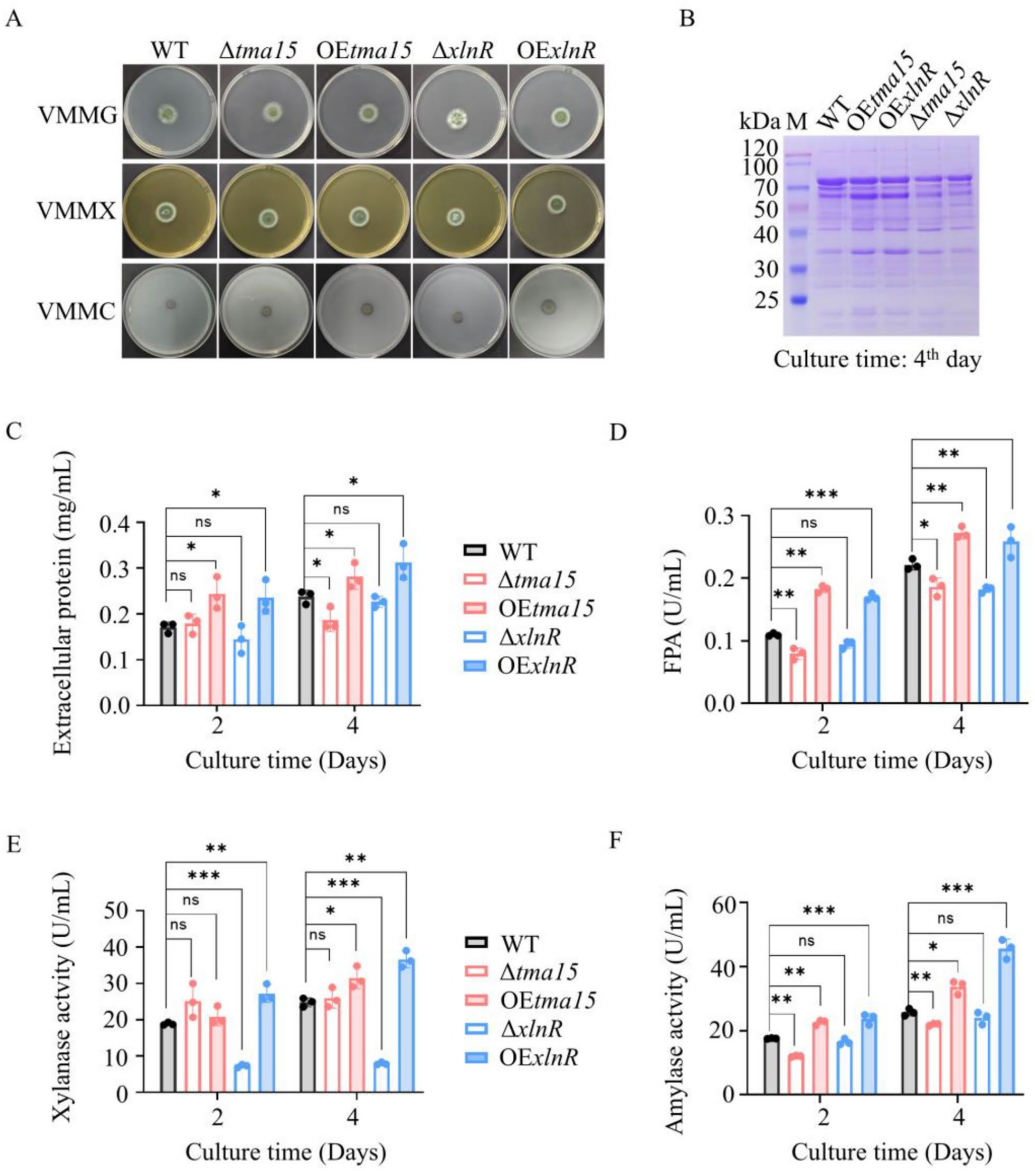
Colony morphology and enzymatic activity assay of the WT strain and various mutants. **A** The colony morphology of strains cultivated on VMM added with 2% glucose (VMMG), 2% xylose (VMMX), and 2% cellulose (VMMC) agar plate. The strains were cultivated at 30 °C for 6 days. **B** SDS-PAGE of supernatants from different strains. **C** The extracellular protein concentration.** D** Filter paper activity (FPA). **E** Xylanase activity. **F** Amylase activity. *p < 0.05, **p < 0.01, ***p < 0.001

We constructed the Po*tma15* gene deletion strain (Δ*tma15*) and overexpression strain (OE*tma15*) to investigate the biological role of the PoTma15 protein. We then compared these mutants (Δ*tma15* and OE*tma15*) to the Po*xlnR* gene deletion mutant (Δ*xlnR*) and Po*xlnR* gene overexpression mutant (OE*xlnR*) that were previously reported [44]. Their colonies were about the same size and exhibited an identical greenish-brown color when the strains were cultivated on VMMG or VMMX (VMM added with 2% xylose) agar (Fig. 3A, left). When the mutants were grown on VMMC agar, the colonies of strains WT, OE*tma15*, and OE*xlnR* were large and had cellulolytic halo surrounding them*,* indicating that the strains can grow well on cellulose as a carbon source and had a good ability to secrete cellulase. In contrast, the colonies of strains Δ*tma15* and Δ*xlnR* are small without cellulolytic halo*,* indicating their limited ability to secrete cellulase and use cellulose as a carbon source (Fig. 3A).

For the VMMC agar plate, pure cellulose served as the sole carbon source. The cellulase production was more effectively induced by complex carbon sources derived from plant materials than pure cellulose. Thus, we cultured the WT strain and the mutants in a submerged medium supplemented with cellulose and wheat bran. Compared to the WT strain, the OE*tma15* and OE*xlnR* mutants showed noticeably darker protein bands, particularly in the 50 ~ 80 kDa range (Fig. 3B). Then, the extracellular protein production and filter paper activity (FPA) representing the overall cellulase activity, xylanase activity, and amylase activity were assayed (Fig. 3C–F). Compared to the WT strain, OE*tma15* and OE*xlnR* mutants showed higher extracellular protein production, whereas Δ*tma15* showed lower extracellular protein production (Fig. 3C). Compared to the WT strain, the OE*tma15* and OE*xlnR* mutants showed higher FPAs, whereas Δ*tma15* and Δ*xlnR* showed lower FPAs (Fig. 3D). The Δ*xlnR* showed significantly lower xylanase production. In contrast, the OE*xlnR* showed significantly higher xylanase production. The xylanase production of Δ*tma15* mutant is similar to that of WT strain. On the 4th day of cultivation, the xylanase production of OE*tma15* was higher than that of the WT strain (Fig. 3E). Compared to the WT strain, Δ*tma15* showed lower amylase production, whereas OE*tma15* and OE*xlnR* showed significantly higher amylase production. The amylase production of Δ*xlnR* mutants is similar to that of the WT strain (Fig. 3F). The results demonstrated that the PoTma15 protein positively correlates to extracellular cellulase. The increase in cellulase, xylanase, and amylase production contributed to the increased extracellular protein production due to Po*tma15* overexpression.

### PoTma15 is not directly associated with the transcription of cellulase genes

The extracellular proteins of *P. oxalicum*, primarily composed of glycoside hydrolases such as cellulases, hemicellulases, and amylases [5], are mainly controlled at the transcriptional level [8]. PoTma15, identified through a putative interaction with the transcription factor PoXlnR and positively related to cellulase production, was initially hypothesized to play a role in regulating the transcription of these genes.

**Fig. 4:**
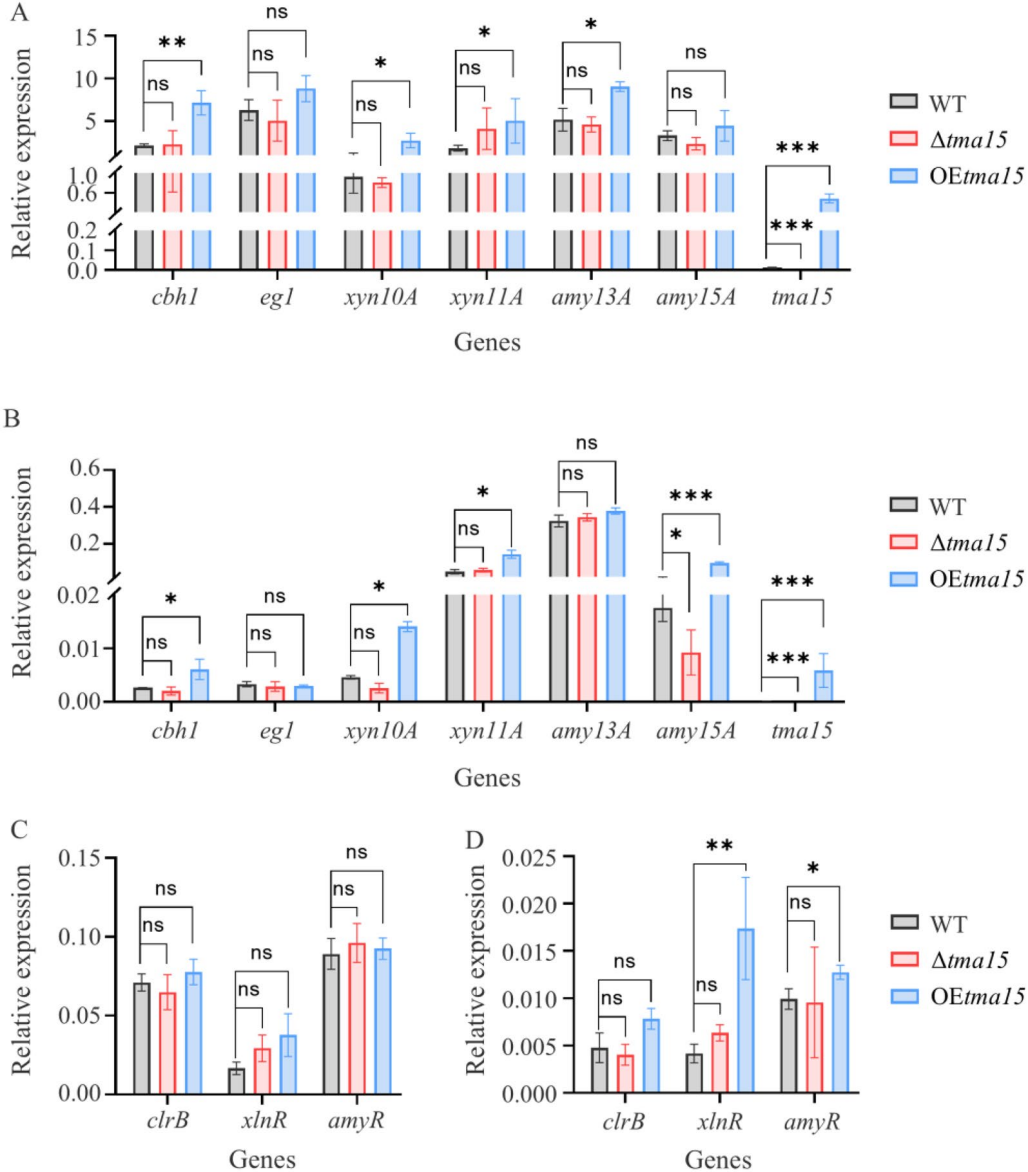
Determination of the transcription levels of the Po*tma15* gene, extracellular glycoside hydrolase-encoding genes, and transcription factor-encoding genes in WT, Δ*tma15*, and OE*tma15*. Transcription levels of the Po*tma15* gene and extracellular glycoside hydrolase-encoding genes when the strains were cultivated in VMMC (**A**) and VMMG (**B**) liquid, respectively. Transcription levels of transcription factor-encoding genes when the strains were cultivated in VMMC (**C**) and VMMG (**D**) liquid, respectively. The values were normalized to the *actin* gene levels considered 1. *p < 0.05, **p < 0.01, ***p < 0.001

The transcription levels of Po*tma15* gene, major cellulase encoding gene (cellobiohydrolase gene *cbh1*/*cel7A* and endo-β-1,4-glucanase gene *eg1*/cel7B), major hemicellulase encoding gene (xylanase genes *xyn10A* and *xyn11A*), major amylase-encoding genes (*amy13A* and *amy15A*), and three key transcription factor-encoding genes (*clrB*, *xlnR*, and *amyR*) were assayed in the WT strain, and Δ*tma15* and OE*tma15* mutants when the strains were cultivated in VMMC and VMMG liquid, respectively (Fig. 4). The transcription level of the Po*tma15* gene in the OE*tma15* mutant is significantly higher than that in the WT strain in both VMMC and VMMG liquid (Fig. 4A, B).

When the strains were cultivated in VMMC liquid, the transcription levels of cellulase genes *cbh1* and *eg1*, hemicellulase genes *xyn10A* and *xyn11A*, and amylase genes *amy13A* and *amy15A* in the Δ*tma15* mutant were not significantly different from those in the WT. The transcription levels of *eg1* and *amy15A* in the OE*tma15* mutant did not differ significantly from those of the WT strain. The transcription levels of *cbh1*, *xyn10A*, *xyn11A*, and *amy13A* in the OE*tma15* mutant were found to be slightly elevated in comparison to the WT, exhibiting 2.4-, 2.0-, 2.6-, and 0.75-fold increases, respectively (Fig. 4A). Deleting the Po*tma15* gene did not affect the transcription levels of major (hemi)cellulase and amylase genes. Furthermore, there was no significant difference in the transcription levels of the three transcription factor-encoding genes (*clrB*, *xlnR*, and *amyR*) among the WT strain, Δ*tma15* and OE*tma15* mutants (Fig. 4C). When the strains were cultured in VMMG liquid, the transcription level of six extracellular glycoside hydrolase genes and three transcription factor genes are generally lower than that in VMMC (Fig. 4B and D). The transcription levels of *cbh1*, *xyn10A*, *xyn11A*, *amy15A*, *xlnR*, and *amyR* in the OE*tma15* mutant were found to be slightly elevated in comparison to the WT, exhibiting 1.2-, 2.0-, 1.9-, 4.4-, 3.1- and 0.2-fold increases, respectively. Similar to the results of the strain cultivated in VMMC, deleting the Po*tma15* gene did not affect the transcription levels of major (hemi)cellulase genes and three transcription factor-encoding genes (*clrB*, *xlnR*, and *amyR*) (Fig. 4B and D).

These results disproved our initial hypotheses. The OE*tma15* mutant showed only slight upregulation of cellulase genes, contrasting the typical hundreds or thousands of folds of upregulation observed when transcriptional activators like ClrB and XlnR were overexpressed [12]. Furthermore, deleting the Po*tma15* gene did not affect the transcription levels of major (hemi)cellulase and amylase genes. The results indicated that the PoTma15 protein might not be directly associated with transcription regulation.

### PoTma15 is associated with the translation machinery

Since PoTma15 has little to do with cellulase gene transcription, by what biological process does PoTma15 affect cellulase production? The Tma15-TAP strain was generated by fusing the TAP tag (HA-FLAG) at the C-terminus of PoTma15. The WT strain 114–2 containing native PoTma15 was used as a control. The biological triplicates were used for the TAP–MS experiment. After two-step tandem purification, the final eluate was divided into three sections: one for SDS-PAGE, one for Western blot, and one for MS–MS assay to identify the putative interacting proteins of the PoTma15 bait, respectively.

**Fig. 5:**
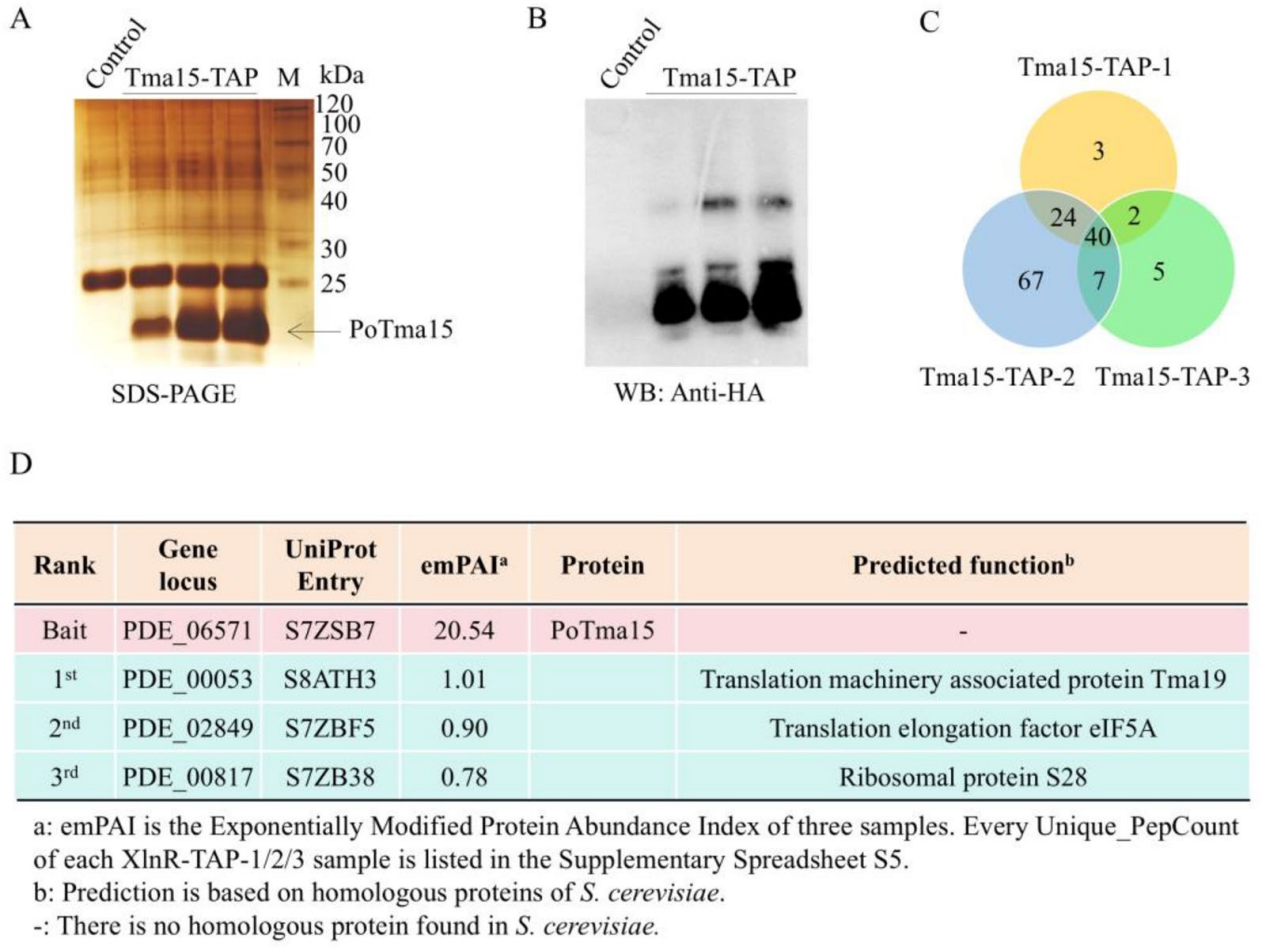
The results of TAP–MS using PoTma15 as bait.** A** Silver staining after SDS-PAGE of Tma15-TAP eluate. **B** Western blot of Tma15-TAP eluate using anti-HA antibody. **C** Intersection proteins among three samples Tma15-TAP-1/2/3. **D** The top three proteins that have putative interactions with PoTma15 identified by TAP–MS. The detailed information, including Unique_PepCount identified by LC–MS/MS, gene locus, theoretical PepCount, emPAI, and predicted functions of putative interacting proteins, are shown in Supplementary Spreadsheet S6

The theoretical molecular weight of the PoTma15 protein is 14.7 kDa. One specific band of about 14 kDa was found between the control (WT) and Tma15-TAP from the gel of SDS-PAGE (Fig. 5A, black arrow). The band was sliced from the gel and identified by LC/MS–MS as the PoTma15 with the highest score, emPAI, and coverage (Supplementary Spreadsheet S5). Western blot analysis also indicated the existence of the PoTma15 bait with a strong signal (Fig. 5B).

The third section of the eluent was analyzed by LC–MS/MS to identify the PoTma15 bait and its putative interacting proteins. The PepCount identified in biological triplicates (Tma15-TAP-1, Tma15-TAP-2, and Tma15-TAP-3) through TAP–MS are listed in Supplementary Spreadsheet S6 (Sheets: Tma15-TAP-1/2/3). The proteins observed in all three XlnR-TAP-1/2/3 samples, but not in the controls are listed in Supplementary Spreadsheet S6 (Sheet: Intersection of three samples). The proteins were ranked by emPAI [41]. 69, 131, and 54 proteins were identified in Tma15-TAP-1, Tma15-TAP-2, and Tma15-TAP-3, respectively. 40 proteins observed in all three Tma15-TAP samples are considered credible proteins that have putative interaction with PoTma15 (Fig. 5C). The top three proteins are shown in Fig. 5D.

As expected, the bait PoTma15 has the highest emPAI among the proteins identified by LC–MS/MS. Among the other proteins, PDE_00053 (UniProt Entry S8ATH3), an ortholog of *S*. *cerevisiae* Tma19p (36.4% identity), exhibited the highest emPAI. Tma19p is a translation-machinery-associated protein that interacts with ribosomes [45]. The protein with the second-highest emPAI is PDE_02849 (Uniprot Entry S7ZBF5), an ortholog of *S*. *cerevisiae* translation elongation factor eIF5A (74.2% identity), which binds to translation machinery components and affects translation elongation in yeast [46]. The protein with the third-highest emPAI is PDE_00817 (Uniprot Entry S7ZB380), an ortholog of *S. cerevisiae* ribosomal protein S28 (91.2% identity), which is the component of the small ribosomal subunit. These results indicated that the PoTma15 protein is associated with the translation machinery.

There was no PoXlnR among the putative interacting proteins of the PoTma15 bait. Since the putative collaborators identified by TAP–MS contain both the true direct interactors of the bait protein and the proteins that indirectly interact with the bait protein mediated by other proteins, we conducted a yeast two-hybrid (Y2H) assay to ascertain whether PoTma15 directly interacted with PoXlnR. Y2H experiments did not reveal any direct physical interaction between PoTma15 and PoXlnR. On the quadruple dropout (QDO, SD–Ade/–His/ − Leu/ − Trp) with X-α-gal, the positive control shows blue, while the hybridized strains were not colored (Fig. S2). This result indicated that there was no direct interaction between PoTma15 and PoXlnR.

Then, we analyzed the results of XlnR-TAP and Tma15-TAP and found overlaps between their putative interacting proteins. For example, two ribosomal proteins, PDE_05108 and PDE_05315, are present in all six samples of XlnR-TAP-1/2/3 and Tma15-TAP-1/2/3 (Supplementary Spreadsheet S3 & S6, Sheet: Intersection of three samples). Tma19, eIF5A, and ribosomal protein S28, which are the top three proteins related to PoTMA15 (Fig. 5D), do not present in all three samples of XlnR-TAP-1/2/3, but do present in two of them, respectively: Tma19 in XlnR-TAP-1/2, eIF5A in XlnR-TAP-1/3, and S28 in XlnR-TAP-2/3 (Supplementary Spreadsheet S3, Sheets: XlnR-TAP-1/2/3). Findings from Y2H, Tma15-TAP, and XlnR-TAP raise the possibility that PoTma15 and PoXlnR indirectly associate via translation machinery components.

### The Δ*tma15* mutant showed resistance to the translation-inhibiting effects of cycloheximide

The result of Tma15-TAP showed that the top three putative interactors with PoTma15 include two components (eIF5A and 40S ribosomal protein) of the translation machinery and one translation-machinery-associated protein (Tma19), which gave a hint that PoTma15 is the protein associated with the translation machinery. To ascertain whether PoTma15 function is related to the translation, cycloheximide (CHX), an inhibitor of eukaryotic translation elongation [47], was added to the basic medium culture of WT, Δ*tma15*, and OE*tma15*, respectively, to observe its effect on the protein production.

**Fig. 6:**
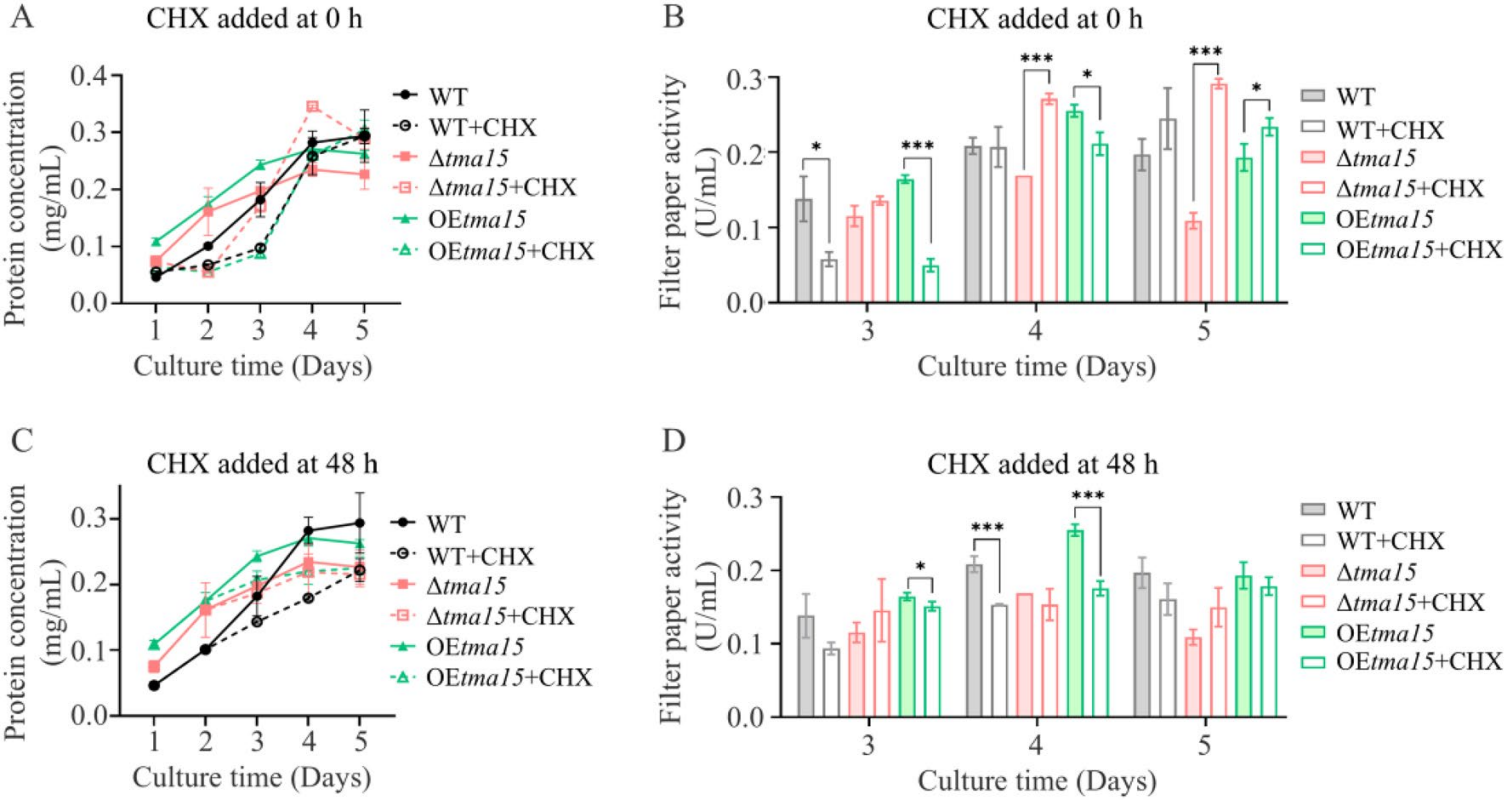
Effect of cycloheximide (CHX) on extracellular protein and cellulase production on the WT, Δ***tma15***, and OE***tma15***. The extracellular protein production (**A**) and cellulase production (**B**) of different strains when CHX was added during medium preparation (0 h). The extracellular protein production (**C**) and cellulase production (**D**) of different strains when CHX was added to the medium at the culture time of 48 h. *p < 0.05, **p < 0.01, ***p < 0.001

We used “strain + CHX” to indicate the strains grown in a medium containing CHX in Fig. 6. CHX was added to the medium with final concentration of 20 µg/mL during medium preparation (0 h) or after a 2-day culture (48 h). When WT + CHX and OE*tma15* + CHX were cultivated in a medium containing CHX (0 h), their extracellular protein production was significantly lower during the first 4 days of incubation. It did not reach the levels of WT and OE*tma15* until the 4th day in comparison to strains WT and OE*tma15* grown in a medium without CHX. For example, the extracellular protein production of WT + CHX and OETma15 + CHX was only 53.6% and 36.2% of those of WT and OE*tma15*, respectively, on the 3rd day of culture. The extracellular protein production of Δ*tma15* + CHX was inhibited less than those of WT + CHX and OE*tma15* + CHX. The extracellular protein production of Δt*ma15* + CHX was 86% of that of Δ*tma15* on the 3rd day of culture; it was even higher than that of Δ*tma15* on the 4th and 5th days of culture (Fig. 6A). There was a similar pattern in the FPAs produced by the strains. The FPAs of OE*tma15* + CHX and WT + CHX were only 30.3% and 42.0% of WT and OE*tma15*, respectively, on the 3rd day of culture. The FPA of Δ*tma15* + CHX was even higher than that of Δ*tma15* (Fig. 6B).

We also assayed the protein concentrations and FPA when CHX was added at the culture time of 48 h. The extracellular protein production of WT + CHX and OE*tma15* + CHX was significantly inhibited compared to WT and OE*tma15*. For example, the extracellular protein production of WT + CHX and OE*tma15* + CHX decreased to 63.5% and 71.2% of those of WT and OE*tma15*, respectively, on the 4th day of culture. The extracellular protein production of Δ*tma15* + CHX was essentially unaffected compared to the Δ*tma15* mutant (Fig. 6C). There was a similar pattern in the FPAs produced by the strains. The FPAs of OE*tma15* + CHX and WT + CHX were 73.5% and 68.8% of WT and OE*tma15*, respectively, on the 4th day of culture. The FPA of Δ*tma15* + CHX was essentially unaffected in comparison to Δ*tma15* (Fig. 6D).

The results demonstrated that in comparison to WT and OE*tma15*, the production of total protein and extracellular cellulase was less affected by CHX in the Δ*tma15* mutant if the biomass differences of each mutant are not taken into account*,* suggesting a stronger resistance to the translation-inhibiting effects of cycloheximide in this mutant.

## Discussion

PoTma15 was discovered in the PoXlnR-TAP results. Initially, we hypothesized that it could be a protein that directly interacts with PoXlnR or functions as a cofactor that aids PoXlnR in triggering the transcription of cellulase genes in the nucleus. However, subsequent studies supported the idea that PoTma15 is closely related to the translation rather than the transcription process.

We observed that the function of the PoTma15 protein was unaffected by the addition of GFP or TAP tags. The growth of Tma15-GFP and Tma15-TAP on VMMG and VMMX agar and cellulase production in VMMC liquid were almost identical to those of the WT (Fig. S3). However, an interesting result is that the XlnR-TAP strain showed higher cellulase production than the WT (Fig. S3B). In the XlnR-TAP strain, the XlnR protein fused with a TAP tag at the C-terminus substitutes the native XlnR protein. The result suggested that XlnR proteins fused with a TAP tag stimulated cellulase production more than native XlnR. In *T. reesei*, the activation effect of Xyr1 on target genes is directly associated with its C-terminal polypeptide. The C-terminal residues 861–940 mediate the homodimerization of Xyr1, which is essential for the transcription of the cellulolytic gene [48]. A single point mutation A824V can render inducer-independent transcription of cellulolytic genes [49]. In *P. oxalicum*, mutation of the corresponding alanine residue (A871) in PoXlnR to valine, isoleucine, and tyrosine increased the production of xylanases and cellulases [50]. Another A873Y mutant enabled significantly enhanced production of xylanolytic enzymes in the medium with cellulose as the carbon source [18]. These findings indicated that the C-terminal polypeptide is important for the activating roles of Xyr1/XlnR. Therefore, it is assumed that adding additional amino acids of TAP tag affected the function of PoXlnR.

The relationship between PoTma15 and PoXlnR is worth exploring. Transcription factors can only activate or repress gene transcription after binding to their target genes in the nucleus [51]. PoTma15 was found in the XlnR-TAP results. This finding raises the question of why the putative interactor of a transcription factor is localized in the cytoplasm. PoXlnR is a Zn2Cys6 binuclear zinc cluster transcription factor. Although many Zn2Cys6-type proteins were commonly thought localized within the nucleus on a constitutive and permanent basis [52], studies have also shown that some transcription factors, such as AflR and NirA in *A. nidulans* and Seb1 in *Aspergillus fumigatus*, do not behave this way: they also disperse in the cytoplasm [53–55]. Cellulase gene transcription in *T. reesei* was demonstrated to stimulate de novo biosynthesis of TrXyr1 in the cytoplasm and its nuclear import [56]. *A. niger* AnXlnR has also been reported to be cytoplasmic in its inactive state and nuclear import under inducing conditions [57]. Thus, the putative interacting proteins identified in XlnR-TAP could comprise not only proteins associated with PoXlnR in the nucleus—but also proteins associated with PoXlnR de novo synthesis in the cytoplasm, and potentially proteins that are indirectly associated with XlnR through other protein bridges. In fact, in addition to translation-associated proteins like PoTma15 and ribosomal protein L38, we observed nuclear proteins, such as Rco1/RcoA, also known as Tup1 (PDE_01024, Supplementary spreadsheet S3, Sheet:Intersection of three samples, row 52) and Ssn6, also known as Cyc8 (PDE_03177, Supplementary spreadsheet S3, Sheet:Intersection of three samples, row 138). Tup1 and Cyc8 form the Tup1–Cyc8 complex, known to be localized in the nucleus and required for TrXyr1 activation [58]. PoTma15 is located in the cytoplasm, and the top overlapping proteins in XlnR-TAP and Tma15-TAP samples have translationally relevant properties. Based on the above results it was speculated that PoTma15 was captureded in the XlnR-TAP sample becaused it is associated with the PoXlnR de novo protein synthesis machinery in the cytoplasm.

The top two proteins associated with PoTma15 are eIF5A and Tma19, whose homologous proteins in *S. cerevisiae* are also found in the cytoplasm [59, 60]. eIF5A was formerly erroneously believed to be an initiation factor [61]. Now, eIF5A has been actually identified as a translation elongation factor that stimulates translation elongation [46, 60]. eIF5A depletion caused an increase in the average ribosome transit time and a significant decrease in total protein synthesis [46, 62]. Tma19 is a highly conserved eukaryotic protein generally called TCTP (translationally controlled tumor protein), which also correlates to translation elongation as it was identified as a regulator of the translation elongation factor eEF1A [59, 63]. The deletion of Tma19 decreased the protein synthesis rates in yeast [45]. Therefore, we hypothesize that PoTma15 may be associated with translational elongation, given that both eIF5A and Tma19 are linked to translational elongation.

The results of the cycloheximide addition experiment also support the association of PoTma15 with translation elongation. The results showed that the Δ*tma15* mutant had stronger resistance to cycloheximide than the WT and OE*tma15.* The result was not surprising, as cycloheximide is a translation elongation inhibitor by cycloheximide binding the ribosome and inhibiting eEF2-mediated translocation [47]. Many studies have revealed that cycloheximide resistance resulted from the absence, disruption, or mutation of some translation machinery components. For example, the absence of ribosomal protein L24 and mutations of ribosomal proteins L41 and L29 caused increased resistance to cycloheximide [64–66]. The loss of four ribosomal protein methyltransferases, which are important for ribosome biogenesis and translation elongation fidelity, also exhibited resistance to cycloheximide [67].

The translation machinery contains a large number of components. Among the 39 putative interacting proteins of PoTma15, there are 3 other proteins (Supplementary spreadsheet S6, row12/16/17) associated with the translation machinery in addition to the top 3 proteins (Tma19, eIF5A, and ribosomal protein S28). Although the results of Tma15-TAP and cycloheximide addition experiments showed the association between PoTma15 and the translation machinery, it will take more in-depth investigation to ascertain which translation machine component PoTma15 directly interacts with and the exact mechanism by which its absence causes resistance to cycloheximide.

Modulation of specific translation machinery components can affect translation efficiency and protein production. For example, the elimination of ribosome preservation factor Stm1, which interacts with ribosomes and affects the interaction of translation elongation factor eEF3 with ribosomes, improved the translation efficiency of *S. cerevisiae* [68, 69]. Translational elongation factor eEF1A has also been used as an effective target to increase cellulase production [32]. As PoTma15 is involved in the production of cellulase and the production of total extracellular proteins, it can not only be used to widen the pathway of cellulase production but possibly serve as engineering targets for using filamentous fungi as cell factories in the future to produce other heterologous proteins or bioproducts.

## Materials and methods

### Fungal strains and culture conditions

*P. oxalicum* 114-2 wild type (WT) (CGMCC 5302), which was previously classified as *Penicillium decumbens* [5], was used as the original parent strain for the construction of mutants in this study. The WT strain and mutants were grown on agar containing 10% wheat bran juice for 5 days at 30◦C to conidiation. For mycelial growth, the strains were grown at 30◦C in 1 × Vogel’s minimal medium (VMM) (50 × Vogel’s salt: 125.0 g Na_3_citrate‧2H_2_O, 250.0 g KH_2_PO_4_, 100.0 g NH_4_NO_3_, 10.0 g MgSO_4_‧7H_2_O, 5.0 g CaCl_2_‧2H_2_O, 0.25 mg biotin, 0.25 g citric acid, 0.25 g ZnSO_4_‧7H_2_O, 0.05 g Fe(NH_4_)_2_(SO_4_)_2_‧6H_2_O, 12.5 mg CuSO_4_‧5H_2_O, 2.5 mg MnSO_4_‧H_2_O, 2.5 mg H_3_BO_3_, 2.5 mg Na_2_MoO_4_‧2H_2_O, and 1 L of water) [70], plus 2% glucose (w/v) (VMMG), 2% xylose (VMMX), or 2% ball-milled cellulose (VMMC) as a sole carbon source. 1.5% agar was added to the VMMG, VMMX, or VMMC for solid culture in plates.

### Construction of different mutants

All primers used in this study are listed in Supplementary Spreadsheet S7.

The construction strategy and verification for the XlnR-TAP strain, which has PoXlnR fused with a FLAG-HA tag at the C-terminus, are shown in Supplementary Fig. S4. For constructing the XlnR-TAP strain, primer pairs XlnR-TAP-UF/XlnR-TAP-UR and XlnR-TAP-DF/XlnR-TAP-DR were used to amplify the 5’-upstream and 3’-downstream homologous arms of the Po*xlnR* gene using *P. oxalicum* WT genomic DNA as the template. Primer pairs hph-F/hph-R were used to amplify the marker gene hygromycin B (*hph*) from the plasmid Psilent1 [71]. Then, the three PCR fragments (5’—upstream and 3’ downstream regions of the Po*xlnR* gene and *hph*) were fused with fusion PCR. The PCR product was then amplified using nested primers XlnR-TAP-CSF/XlnR-TAP-CSR and transformed into the WT to obtain the XlnR-TAP mutant. The proper integration of the FLAG-HA tag was verified by sequencing.

The construction strategy and verification for the Tma15-TAP strain, which has PoTma15 fused with a FLAG-HA tag at the C-terminus, are shown in Supplementary Fig. S5A ~ C. For constructing the Tma15-TAP strain, primer pairs Tma15-TAP-UF/Tma15-TAP-UR and Tma15-TAP-DF/Tma15-TAP-DR were used to amplify the 5’-upstream and 3’-downstream homologous arms of the Po*tma15* gene using *P. oxalicum* WT genomic DNA as the template. Primer pairs hph-F/hph-R were used to amplify the marker gene hygromycin B (*hph*) from the plasmid Psilent1 [71]. Then, the three PCR fragments (5’-upstream and 3’-downstream regions of the Potma15 gene and *hph*) were fused with fusion PCR. The PCR product was then amplified using nested primers Tma15-TAP-CSF/Tma15-TAP-CSR and transformed into the WT to obtain the Tma15-TAP mutant.

The construction strategies and verification for the Δ*tma15* mutant (the gene Po*tma15* was deleted) and OE*tma15* mutant (overexpression of the Po*tma15* gene under the promoter of glyceraldehyde-3-phosphate dehydrogenase *gpdA*) are shown in Supplementary Fig. S5D, E. For constructing the Δ*tma15* mutant, primer pairs Δtma15-UF/Δtma15-UR and Δtma15-DF/Δtma15-DR were used to amplify the 5’-upstream and 3’-downstream homologous arms of the Po*tma15* gene (PED_06571) using *P. oxalicum* WT genomic DNA as the template. Primer pairs hph-F/hph-R were used to amplify the marker gene *hph*. Then, the three PCR fragments (5’- and 3’-flanking regions of the Po*tma15* gene and *hph*) were fused with fusion PCR. The fused product was then amplified using nested primers Δtma15-CSF/Δtma15-CSR and transformed into the WT to obtain the Δ*tma15* mutant.

For constructing the OE*tma15* mutant, the promoter of *A*. *nidulans gpdA* gene (encoding glyceraldehyde-3-phosphate dehydrogenase), a strong constitutive promoter, was fused with the encoding sequence of PoTma15. Primer pairs gpdA-F and gpdA-R were used to amplify the *gpdA* promoter from *A. nidulans*. Primer pairs OEtma15F/OEtma15R were used to amplify the coding and termination sequences of Po*tma15*. Primer pairs hph-F/hph-R were used to amplify the marker gene hygromycin B (*hph*) from the plasmid Psilent1. Then, *gpdA*, coding and termination sequences of Po*tma15*, and the *hph* marker were fused by fusion PCR. The fused product was then amplified using nested primers OEtma15-CSF/OEtma15-CSR and was transformed into the WT to obtain the OE*tma15* mutant.

The construction strategies for the Tma15-GFP mutant (the protein PoTma15 fused with a green fluorescent protein (GFP) are shown in Supplementary Figure S5F, G. For constructing the Tma15-GFP strain, primer pairs Tma15-GFP-UF/Tma15-GFP-UR were used to amplify the 5’-upstream and 3’-downstream homologous arms of the Po*tma15* gene using *P. oxalicum* WT genomic DNA as the template. Primer pairs gfp-F/gfp-R were used to amplify the *gfp* gene from the plasmid pEGFP. Primer pairs hph-F/hph-R were used to amplify the marker gene hph from the plasmid Psilent1. Then, the four PCR fragments (5’-upstream and 3’-downstream of the Po*tma15* gene, *gfp,* and *hph*) were fused with fusion PCR. The PCR product was then amplified using nested primers Tma15-GFP-CSF/Tma15-GFP-CSR and transformed into the WT to obtain the Tma15-GFP strain. For constructing the ΔT-Tma15-GFP strain, primer pairs Tma15-GFP-UF/Tma15-GFP-UR and ΔT-Tma15-GFP-DF/Tma15-DR were used to amplify the 5’-upstream and 3’-downstream homologous arms of the Potma15 gene using *P. oxalicum* WT genomic DNA as the template. Primer pairs gfp-F/gfp-R were used to amplify the *gfp* gene from the plasmid pEGFP. Primer pairs ptrA-F/ptrA-R were used to amplify the marker gene *ptrA* from the plasmid Psilent1. Then, the four PCR fragments (5’-upstream and 3’-downstream of the Po*tma15* gene, *gfp*, and *ptrA*) were fused with fusion PCR. The PCR product was then amplified using nested primers Tma15-GFP-CSF/Tma15-GFP-CSR and transformed into the ΔTma15 to obtain the ΔT-Tma15-GFP strain.

The amino acid sequences of PoTma15 homologous proteins from various filamentous fungi were obtained from the UniProt database (https://www.uniprot.org/) through BLASTp using the protein sequence of PoTma15 (PDE_06571, UniProt entry: S7ZSB7) as a query. Multiple sequence alignment and a phylogenetic tree was constructed using the neighbor-joining method via the software MEGA 7.0 [72].

### Fungal colony and microscopic observation

Fresh spore solutions with equal spore concentrations (10^7^/mL) of various strains were prepared. 2 μL of each spore solution was spotted on VMMG, VMMX, or VMMC agar plates. The plates were cultured for 4 days at 30 °C for fungal colony observation. 100μL of fresh spore suspensions of Tma15-GFP strain were spread onto VMMG agar, and several sterile coverslips were inserted into the agar. The Tma15-GFP strain was cultivated at 30 °C for 24 h. Then, the coverslips were taken out, and the mycelia on them were stained for 15 min in the dark using 1 µg/mL Hoechst 33342 to achieve nucleus staining. The coverslips were viewed under a high-sensitivity laser scanning confocal microscope (ZEISS LSM900) (Carl Zeiss, Oberkochen, Germany). The excitation light at 488 nm wavelength was used to observe green fluorescence and at 405 nm for the observation of nuclear staining.

### Determination of cellulase activity and extracellular protein concentration

Fresh spore suspensions of various strains were cultivated in VMMG liquid at 30 °C for 24 h. Then, 0.3 g of filtered hyphae was transferred to 100 mL of VMM added with 1% wheat bran and 1% cellulose (w/v) media and cultivated at 200 rpm and 30 °C for 5 days. The culture supernatants (crude enzyme) were collected by centrifugation to remove cellulose and wheat bran. The extracellular amylase activity was measured using 2% soluble starch ((Sigma-Aldrich, USA) as substrate. The amylase activity of the culture supernatants was assayed as previously described [12]. The following components, 0.5 mL (or diluted) culture supernatants and 1.5 mL 1% starch solution (prepared in 0.2 M acidic buffer, pH 4.8), were added into a 25 mL colorimetric tube. The reaction mixture was incubated at 40 °C for 10 min. Then 3 mL of DNS reagent (10 g 3, 5-dinitrosalicylic acid, 20 g sodium hydroxide, 200 g sodium potassium tartrate, 2.0 g redistilled phenol, and 0.50 g sodium sulfite anhydrous per 1000 mL DNS reagent) was added to stop the reaction. The tubes were placed in boiling water for 10 min, and then 20 mL of distilled water was added. The absorbance was determined at 540 nm. The same DNS method was used for the assay of filter paper activity (FPA) and xylanase activity, using Whatman No. 1 filter paper (GE Healthcare companies, UK) and birch xylan (Ryon, Shanghai, China) as the substrate, respectively, and with the enzymatic hydrolysis time at 50◦C of 60 min and 30 min, respectively. One enzyme activity unit was defined as the amount of enzyme required for producing 1 μmol glucose or 1 μmol xylose per minute under the assayed conditions. Extracellular protein concentrations in the culture supernatant were assayed following the protocols of the Modified Bradford Protein Assay Kit (Sangon Biotech China).

### Tandem affinity purification and mass spectrometry

Each treatment for TAP–MS was performed in three biological replicates. The tandem affinity purification was performed according to the methods previously described by Hu et al. [36]. Fresh conidial suspensions of the *P. oxalicum* WT, XlnR-TAP, or Tma15-TAP strains were inoculated in 2 L of VMMG liquid at 30 ◦C and 200 rpm for 24 h. The mycelia were filtered and washed twice with 0.96% NaCl (w/v) containing 1% DMSO and 1 mM PMSF. Then, the mycelia were ground in liquid nitrogen. The ground powder was transferred to a 100 mL centrifuge tube and added with 15 mL of protein lysis buffer (NaCl 9 g, 1 M Tris–HCl, pH 7.5, glycerin 100 mL, and NP40 1 mL, per 1 L) and 0.05% protease inhibitor cocktail (MedChemExpress, China). After thoroughly mixing the mycelia and lysis buffer, the mycelia was placed on ice for 10 min. The samples were centrifuged at 10,000 rpm and 4 °C for 30 min to obtain the suspension. For the first-step affinity purification, ANTI-FLAG M2 affinity resin (Smart-lifesciences, China) was added to the suspension and incubated overnight at 4 °C with gentle rotation. 3 × FLAG peptide (500 μL, 150 ng/μL) was used to compete with the ANTI-FLAG M2 affinity resin to obtain the first protein eluate. For the second-step affinity purification, ANTI-HA resin (Smart-lifesciences, China) was added to the first protein eluate and incubated overnight at 4 °C with gentle rotation. Finally, 80 μL of 8 M urea was used to elute ANTI-HA resin to obtain the final protein eluate, which contains the putative interacting proteins with the bait protein. The final eluate was divided into three parts. One part of the eluate was separated by 12.5% SDS-PAGE and stained with silver reagent. One part of the eluate was assayed with Western blot using anti-HA as an antibody (ABclonal, China). The other part of the eluate was assayed through LC–MS/MS (APT, Shanghai, China) to determine the putative interacting proteins from the bait protein PoXlnR or PoTma15. Exponentially modified protein abundance index (emPAI) was used to estimate absolute protein amount using the following formula [41]. emPAI = 10^PAI^—1. PAI = N_observed_/N_observable_. N_observed_ is the number of experimentally observed peptides assayed by LC–MS/MS. N_observable_ is the number of theoretically observable tryptic peptides according to the amino acid sequence of each protein.

### Total RNA extraction and gene expression analysis by RT-qPCR

Fresh spore suspensions of various strains were cultivated in VMMG liquid at 30 °C for 24 h. Then, 0.3 g of filtered hyphae was transferred to 50 mL of VMMG or VMMC cultivated at 200 rpm and 30 °C. The mycelia at the culture time of 4- and 24-h were collected via centrifugation and ground in liquid nitrogen. Then, 100 mg of ground powder of each sample was transferred into 1 mL of TRIzol reagent (TaKaRa Biotechnology). Following the manufacturer's instructions, total RNA extraction was performed. cDNA was obtained using a PrimeScript RT Reagent kit with a gDNA Eraser (TaKaRa Biotechnology). For each gene, biological triplicates of the qPCR assay were performed. Light Cycler 480 system with software version 4.0 (Roche, Mannheim, Germany) was used to perform the reaction procedure. The primer pairs for the specific gene Po*mta15*, Po*cbh1*, Poeg1, Po*xyn10A*, Po*xyn11A*, Po*amy13A*, Poa*my15A*, and Po*actin* assayed by qPCR are as follows: Tma15-RT-F/Tma15-RT-R, cbh1-RT-F/cbh1-RT-R, eg1-RT-F/eg1-RT-R, xyn10A-RT-F/xyn10A-RT-R, xyn11A-RT-F/xyn11A-RT-R, amy13A-RT-F/amy13A-RT-R, amy15A-RT-F/amy15A-RT-R, and actin-RT-F/Actin-RT-R. The expression level of a specific gene was based on the control gene Po*actin* (PDE_01092). The outcome of relative expression of the examined gene was calculated as follows: copy number of target gene/actin gene. Statistical significance was considered at P ≤ 0.05. The primers used for qPCR are listed in Supplementary Spreadsheet S7.

### Yeast two-hybrid assay

For yeast two-hybrid assay (Y2H) analysis, the coding sequence (CDS) of truncated PoXlnR (163 to 987 aa) which avoids DNA binding domain of PoXlnR, were amplified by primer pairs XlnR-ADF/XlnR-ADR. Then, the CDS were cloned into the plasmid pGADT7 and transformed into *S. cerevisiae* Y187. The CDS of PoTma15 was amplified by primers Tma15-BDF/Tma15-BDR, cloned into the plasmid pGBKT7, and transformed into *S. cerevisiae* Y2H Gold. Y2H strains were grown on QSD (SD-Leu/ − Trp/-His/ − Ade) agar and QSD/X/Aba (SD-Leu/ − Trp/-His/ − Ade/x-α-gal/Aba) agar to test possible interactions.

## Supplementary Information

Supplementary material 1: Figure S1 Phylogenetic analysis of *P. oxalicum* Tma15 and homologous proteins of various filamentous fungi. The tree was constructed using the Neighbor-Joining method in MEGA 7.0. Alignment gaps and missing data were removed. Statistical confidence of the inferred phylogenetic relationships was evaluated by performing 1000 bootstrap replicates. The tree is depicted to scale, using the same units for branch lengths as the evolutionary distances used to infer the phylogenetic tree.

Supplementary material 2: Figure S2 Interaction analysis between PoTma15 and PoXlnR using yeast two-hybrid assay. The open reading frame (ORF) of PoTma15 was cloned into the plasmid pGBKT7 containing the GAL4 DNA binding domain (DNA-BD). The ORF of the transcription factors PoXlnR was amplified using cDNA from *P. oxalicum* WT as the template and cloned into the plasmid pGADT7 containing the GAL4 activation domain (AD). The plasmid with AD (AD-PoxlnR) was transformed into yeast Y187 to obtain the Y187-AD-XlnR strain. The plasmid with BD (BD-PoTma15) was transformed into yeast Y2H Gold-BD to obtain the Y2H Gold-BD-Tma15 strain. (A) The test of toxicity and autoactivation of the Y187-AD-XlnR strain (left) and Gold-BD-Tma15 strain (right). The results demonstrated that there is no toxicity and autoactivation. (B) The hybridized strains were tested on Quadruple Dropout (QDO, QDO–Ade/–His/−Leu/−Trp) with X-α-gal, where the positive control colony turned blue, and the negative control colony was kept white. The results demonstrated no direct interaction between PoTma15 and PoXlnR.

Supplementary material 3: Figure S3 Colony morphology and filter paper activity assay of the WT strain and various mutants containing GFP or TAP tags. (A) The colony morphology of strains cultivated on VMMG and VMMX agar plate. The strains were cultivated at 30°C for 6 days. (B) Filter paper activity. p < 0.05, **p < 0.01, ***p < 0.001.

Supplementary material 4: Figure S4 Construction strategy and verification of XlnR-TAP strain. (A) Construction strategy of XlnR-TAP strain. (B) Results of diagnostic PCR of XlnR-TAP strain. Lane 1 and lane 2 represent the control *P. oxalicum* WT. Lane 3 (3134 bp) and lane 4 (2881 bp) represent XlnR-TAP. Lane 1 and Lane 3 were amplified using primers xlnR-TAP-UF/hph-YZR. Lane 2 and Lane 4 were amplified using primers hph-YZF/xlnR-TAP-DR. (C) Sequencing results of the protein PoXlnR fused with the TAP (FALG-HA) tag.

Supplementary material 5: Figure S5 Construction strategy and verification of Tma15-TAP, Po*tma15* gene deletion (Δ*tma15*), overexpression (OE*tma15*), and Tma15-GFP strain. (A) Construction strategy of Tma15-TAP strain. (B) Results of diagnostic PCR of Tma15-TAP strain. Lane 1, Lane 2, and Lane 3 represent the control* P. oxalicum* WT. Lane 4 (2074 bp), Lane 5 (3273 bp), and Lane 6 (2803 bp) represent Tma15-TAP. Lane 1 and Lane 4, Lane 2 and Lane 5, and Lane 3 and Lane 6 were amplified using primers Tma15-TAP-UF/hph-YZR, Tma15-TAP-YZF2/Tma15-TAP-YZR2, and hph-YZF/Tma15-DR, respectively. (C) Sequencing results of the protein PoTma15 fused with the TAP (FALG-HA) tag. (D) Construction strategies of strains Δ*tma15* and OE*tma15*. (E) Results of diagnostic PCR of strains Δ*tma15* and OE*tma15*. Lane 1, Lane 2, and Lane 3 represent the control* P. oxalicum* WT. Lane 4 (2376 bp), Lane 5 (2668 bp), and Lane 6 (2803 bp) represent Δ*tma15*. Lane 1 and Lane 4, Lane 2 and Lane 5, and Lane 3 and Lane 6 were amplified using primers ΔTma15-UF/hph-YZR, ΔTma15-YZF/ΔTma15-YZR, and hph-YZF/Tma15-DR, respectively. Lane 7 represents the control* P. oxalicum* WT, and Lane 8 represents the OE*tma15, *amplified using primers OEtma15-YZF/OEtma15-YZR. (F) Construction strategy of Tma15-GFP strain. (G) Results of diagnostic PCR of Tma15-GFP strain. Lane 1 and Lane 2 represent the control *P. oxalicum* WT. Lane 3 (2752 bp) and Lane 4 (2803 bp) represent XlnR-TAP. Lane 1 and Lane 3 were amplified using primers Tma15-GFP-UF/hph-YZR. Lane 2 and Lane 4 were amplified using primers hph-YZF/Tma15-DR.

Supplementary material 6: Spreadsheet S1 Five sliced gels from XlnR-TAP assayed by LC–MS/MS. Sheets “Band 1 ~ Band 5” correspond to the bands ① ~ ⑤ (black arrows) in Fig. 1A.

Supplementary material 7: Spreadsheet S2 Proteins observed in the controls. Sheet:Proteins in controls, the proteins are ranked by Unique PepCount detected by MS. Sheet:MS data, the raw data of MS.

Supplementary material 8: Spreadsheet S3 Proteins interacting with PoXlnR identified through TAP–MS using PoXlnR as the bait. Sheet:Intersection of three samples, proteins observed in the intersection of three XlnR-TAP-1/2/3 samples. Row 4, the bait PoXlnR. Sheets:XlnR-TAP-1/2/3, proteins interacting with PoXlnR identified in sample XlnR-TAP-1/2/3, respectively. The proteins are ranked by exponentially modified protein abundance index (emPAI).

Supplementary material 9: Spreadsheet S4 The homologous proteins of PoTma15 in various filamentous fungi.

Supplementary material 10: Spreadsheet S5 The sliced gel from Tma15-TAP assayed by LC–MS/MS.

Supplementary material 11: Spreadsheet S6 Proteins interacting with PoTma15 identified through TAP–MS using PoTma15 as the bait. Sheet:Intersection of three samples, proteins observed in the intersection of three Tma15-TAP-1/2/3 samples. Row 4, the bait PoTma15. Sheets:Tma15-TAP-1/2/3, proteins interacting with PoTma15 identified in sample Tma15-TAP-1/2/3, respectively. The proteins are ranked by exponentially modified protein abundance index (emPAI).

Supplementary material 12: Spreadsheet S7 The primers used in this study.

## Data Availability

No datasets were generated or analysed during the current study.
