# Supplementary figures and images for: Discovery of a novel translation-machinery-associated protein that positively correlates with cellulase production
Source: Biotechnol Biofuels Bioprod. 2025 Feb 22;18:20. doi: 10.1186/s13068-025-02624-7 (PMC11847360; doi:10.1186/s13068-025-02624-7)

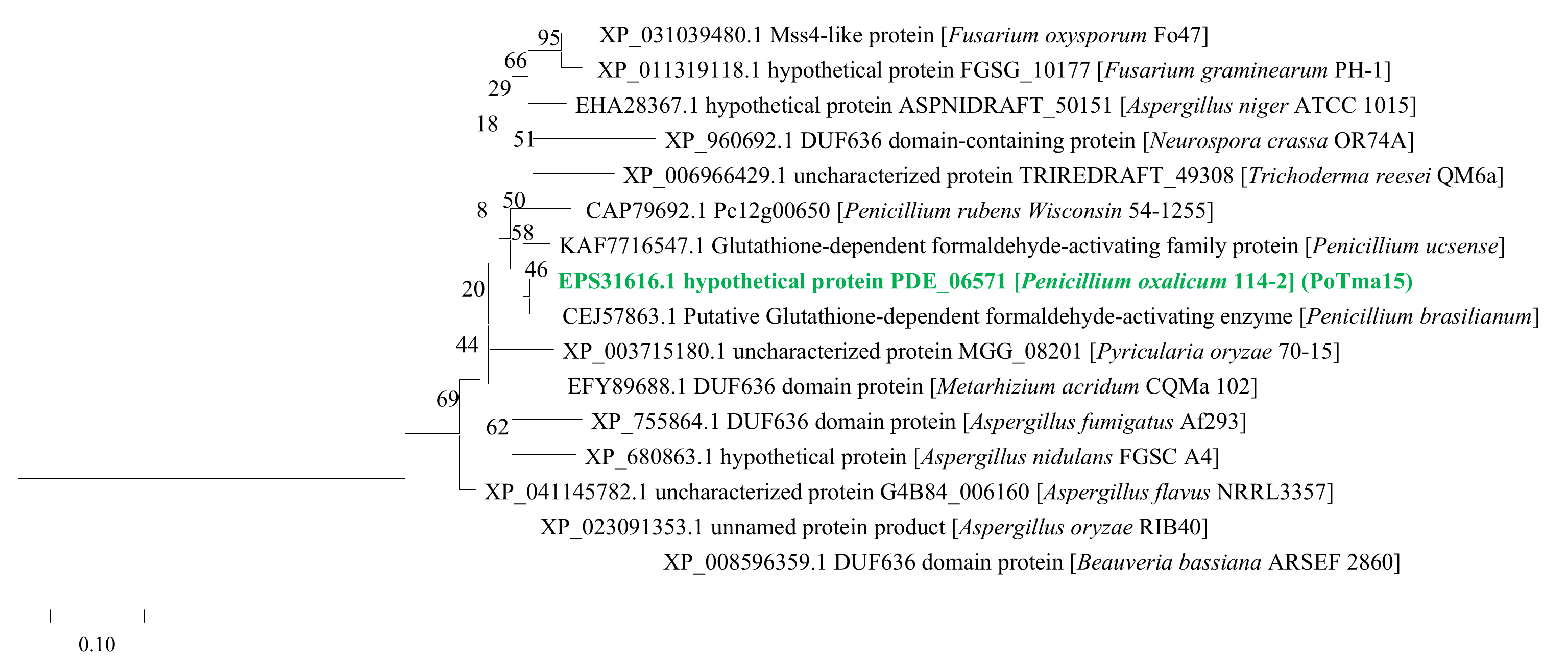

Supplement: Supplementary file 4 — Supplementary material 4: Figure S4 Construction strategy and verification of XlnR-TAP strain. (A) Construction strategy of XlnR-TAP strain. (B) Results of diagnostic PCR of XlnR-TAP strain. Lane 1 and lane 2 represent the control P. oxalicum WT. Lane 3 (3134 bp) and lane 4 (2881 bp) represent XlnR-TAP. Lane 1 and Lane 3 were amplified using primers xlnR-TAP-UF/hph-YZR. Lane 2 and Lane 4 were amplified using primers hph-YZF/xlnR-TAP-DR. (C) Sequencing results of the protein PoXlnR fused with the TAP (FALG-HA) tag. [file 13068_2025_2624_MOESM4_ESM.tif]

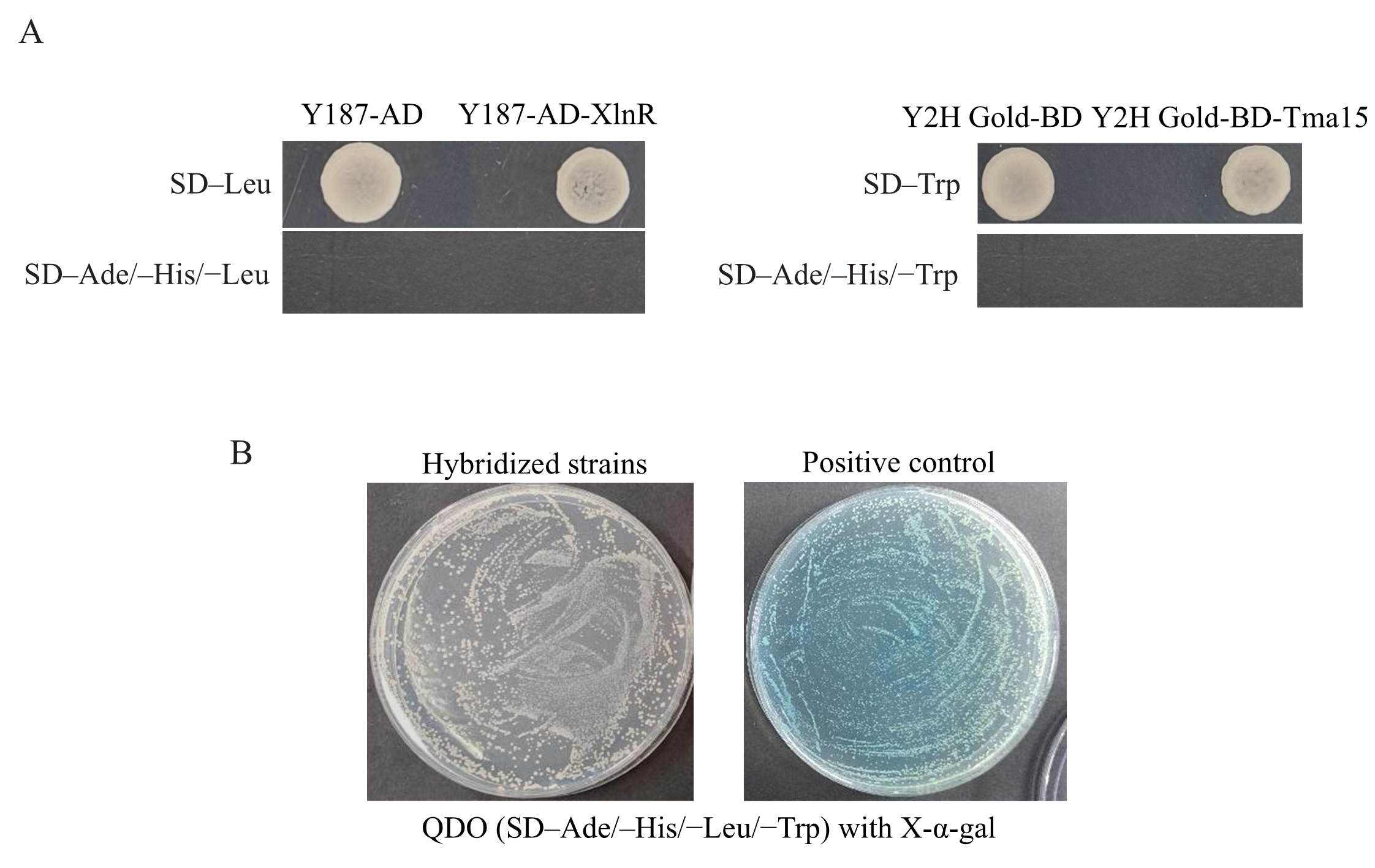

Supplement: Supplementary file 5 — Supplementary material 5: Figure S5 Construction strategy and verification of Tma15-TAP, Potma15 gene deletion (Δtma15), overexpression (OEtma15), and Tma15-GFP strain. (A) Construction strategy of Tma15-TAP strain. (B) Results of diagnostic PCR of Tma15-TAP strain. Lane 1, Lane 2, and Lane 3 represent the control P. oxalicum WT. Lane 4 (2074 bp), Lane 5 (3273 bp), and Lane 6 (2803 bp) represent Tma15-TAP. Lane 1 and Lane 4, Lane 2 and Lane 5, and Lane 3 and Lane 6 were amplified using primers Tma15-TAP-UF/hph-YZR, Tma15-TAP-YZF2/Tma15-TAP-YZR2, and hph-YZF/Tma15-DR, respectively. (C) Sequencing results of the protein PoTma15 fused with the TAP (FALG-HA) tag. (D) Construction strategies of strains Δtma15 and OEtma15. (E) Results of diagnostic PCR of strains Δtma15 and OEtma15. Lane 1, Lane 2, and Lane 3 represent the control P. oxalicum WT. Lane 4 (2376 bp), Lane 5 (2668 bp), and Lane 6 (2803 bp) represent Δtma15. Lane 1 and Lane 4, Lane 2 and Lane 5, and Lane 3 and Lane 6 were amplified using primers ΔTma15-UF/hph-YZR, ΔTma15-YZF/ΔTma15-YZR, and hph-YZF/Tma15-DR, respectively. Lane 7 represents the control P. oxalicum WT, and Lane 8 represents the OEtma15, amplified using primers OEtma15-YZF/OEtma15-YZR. (F) Construction strategy of Tma15-GFP strain. (G) Results of diagnostic PCR of Tma15-GFP strain. Lane 1 and Lane 2 represent the control P. oxalicum WT. Lane 3 (2752 bp) and Lane 4 (2803 bp) represent XlnR-TAP. Lane 1 and Lane 3 were amplified using primers Tma15-GFP-UF/hph-YZR. Lane 2 and Lane 4 were amplified using primers hph-YZF/Tma15-DR. [file 13068_2025_2624_MOESM5_ESM.tif]

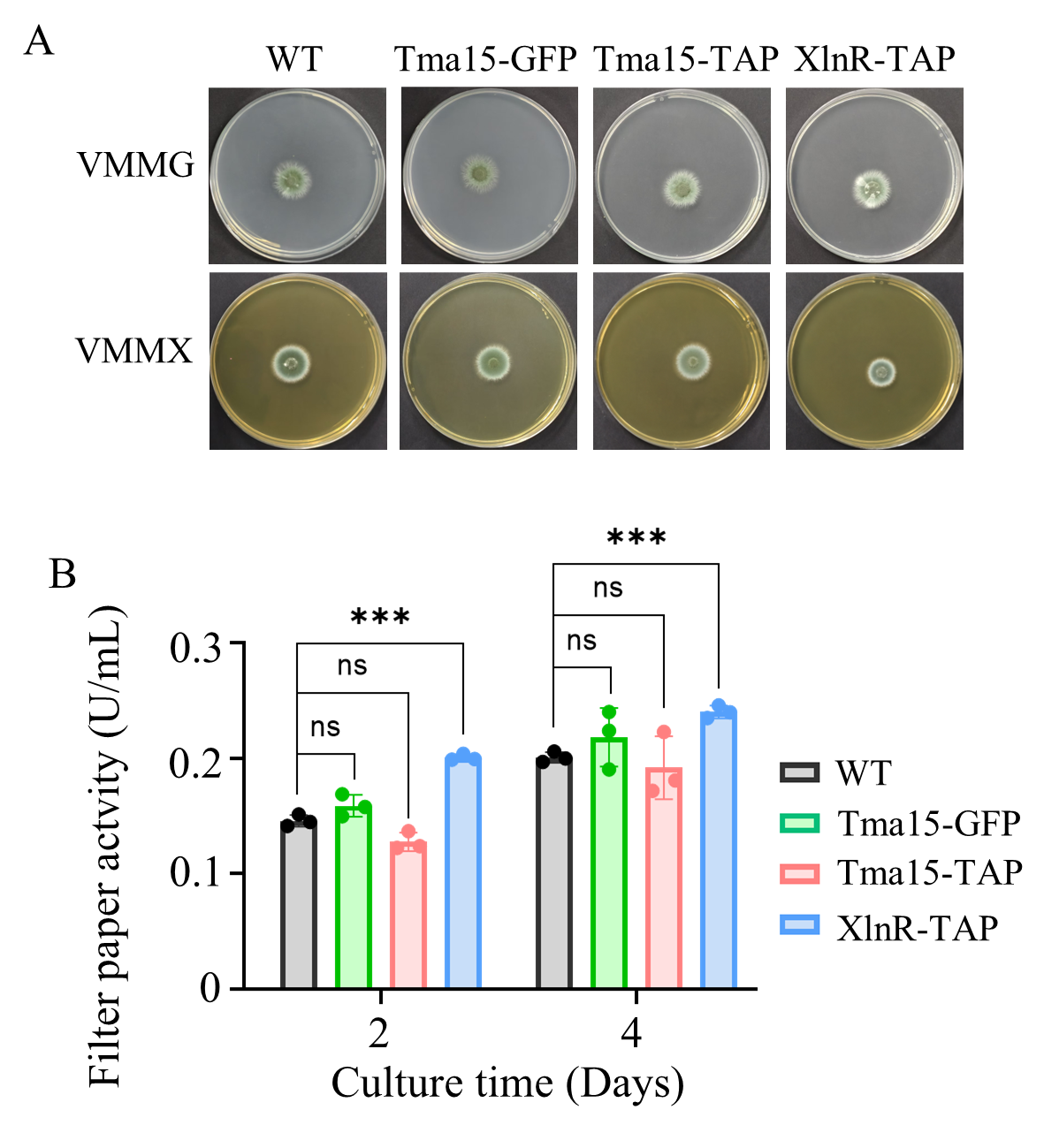

Supplement: Supplementary file 6 — Supplementary material 6: Spreadsheet S1 Five sliced gels from XlnR-TAP assayed by LC–MS/MS. Sheets “Band 1 ~ Band 5” correspond to the bands ① ~ ⑤ (black arrows) in Fig. 1A. [file 13068_2025_2624_MOESM6_ESM.tif]

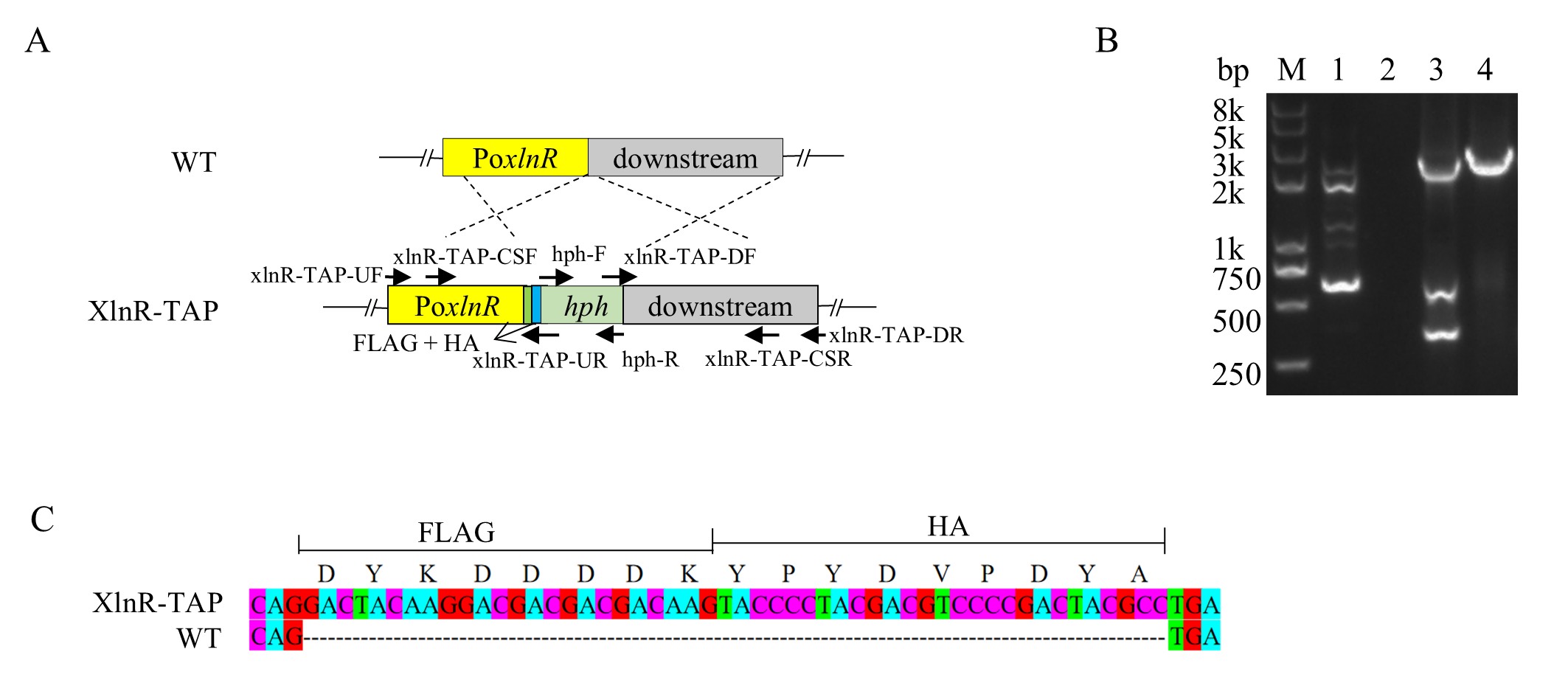

Supplement: Supplementary file 7 — Supplementary material 7: Spreadsheet S2 Proteins observed in the controls. Sheet:Proteins in controls, the proteins are ranked by Unique PepCount detected by MS. Sheet:MS data, the raw data of MS. [file 13068_2025_2624_MOESM7_ESM.tif]

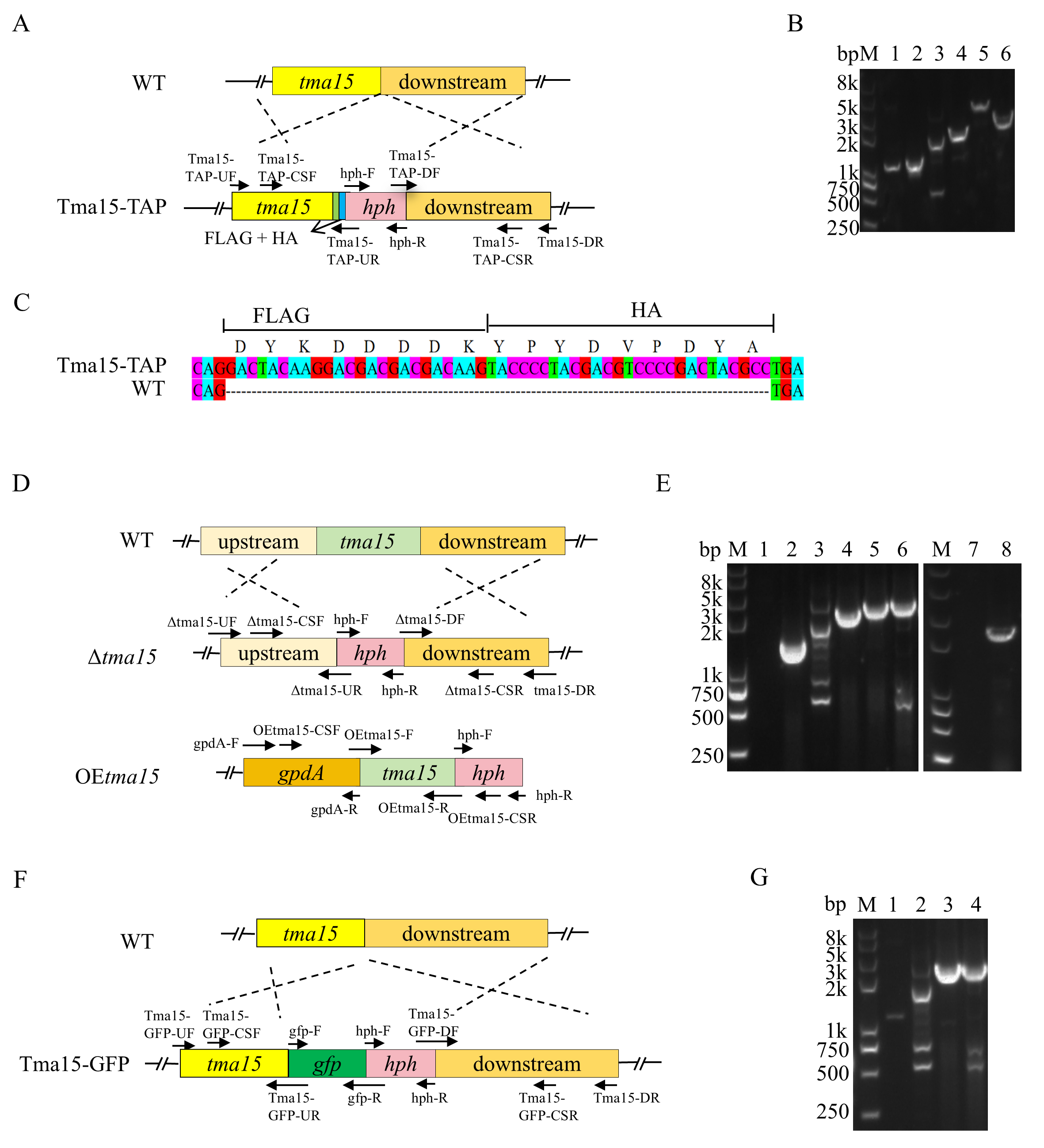

Supplement: Supplementary file 8 — Supplementary material 8: Spreadsheet S3 Proteins interacting with PoXlnR identified through TAP–MS using PoXlnR as the bait. Sheet:Intersection of three samples, proteins observed in the intersection of three XlnR-TAP-1/2/3 samples. Row 4, the bait PoXlnR. Sheets:XlnR-TAP-1/2/3, proteins interacting with PoXlnR identified in sample XlnR-TAP-1/2/3, respectively. The proteins are ranked by exponentially modified protein abundance index (emPAI). [file 13068_2025_2624_MOESM8_ESM.tif]
